# Rapid evolution of cooperation in group-living animals

**DOI:** 10.1186/1471-2148-13-235

**Published:** 2013-10-29

**Authors:** Mathias Franz, Oliver Schülke, Julia Ostner

**Affiliations:** 1 Courant Research Center Evolution of Social Behavior, University of Göttingen, Kellnerweg 6, Göttingen 37077, Germany; 2 Department of Biology, Duke University, Box 90338, Durham, NC 27708, USA

## Abstract

**Background:**

It is often assumed that evolution takes place on very large timescales. Countering this assumption, rapid evolutionary dynamics are increasingly documented in biological systems, e.g. in the context of predator–prey interactions, species coexistence and invasion. It has also been shown that rapid evolution can facilitate the evolution of cooperation. In this context often evolutionary dynamics influence population dynamics, but in spatial models rapid evolutionary dynamics also emerge with constant population sizes. Currently it is not clear how well these spatial models apply to species in which individuals are not embedded in fixed spatial structures. To address this issue we employ an agent-based model of group living individuals. We investigate how positive assortment between cooperators and defectors and pay-off differences between cooperators and defectors depend on the occurrence of evolutionary dynamics.

**Results:**

We find that positive assortment and pay-off differences between cooperators and defectors differ when comparing scenarios with and without selection, which indicates that rapid evolutionary dynamics are occurring in the selection scenarios. Specifically, rapid evolution occurs because changes in positive assortment feed back on evolutionary dynamics, which crucially impacts the evolution of cooperation. At high frequencies of cooperators these feedback dynamics increase positive assortment facilitating the evolution of cooperation. In contrast, at low frequencies of cooperators rapid evolutionary dynamics lead to a decrease in assortment, which acts against the evolution of cooperation. The contrasting dynamics at low and high frequencies of cooperators create positive frequency-dependent selection.

**Conclusions:**

Rapid evolutionary dynamics can influence the evolution of cooperation in group-living species and lead to positive frequency-dependent selection even if population size and maximum group-size are not affected by evolutionary dynamics. Rapid evolutionary dynamics can emerge in this case because sufficiently strong selective pressures allow evolutionary and demographic dynamics, and consequently also feedback between assortment and evolution, to occur on the same timescale. In particular, emerging positive frequency-dependent selection could be an important explanation for differences in cooperative behaviors among different species with similar population structures such as humans and chimpanzees.

## Background

It is commonly assumed that evolution only takes place on very large timescales that include hundreds or thousands of generations. In contrast to this assumption, an increasing number of studies document evolutionary dynamics that occur over just a handful of generations. Well known examples include beak evolution in Darwin finches [1], guppy evolution in response to killifish predation [2] and changes in predator–prey cycles due to algae evolution in response to predation by rotifers [3,4]. An important observation in these studies was that evolutionary dynamics were so fast that they occurred on the same timescale as ecological dynamics, e.g. population dynamics. This opens up the possibility that rapid evolutionary dynamics emerge, i.e. that evolutionary and ecological dynamics feed back on each other on the same timescale. For instance in the case of Darwin’s finches it has been shown that population dynamics were not only driven by changes in food supply and density-dependence but also by evolutionary changes in beak size and shape [1]. Several theoretical and empirical studies on predator–prey interactions, species coexistence and invasion [1,5-12] have shown that the kinds of feedback dynamics that emerge between evolutionary and ecological dynamics can crucially depend on whether evolutionary and ecological dynamics occur on the same timescale.

Rapid evolutionary dynamics can be also important for the evolution of cooperation if evolutionary dynamics affect demographic dynamics. In this context cooperators are individuals who provide benefits to others at some cost to themselves. In contrast, defectors are individuals who do not engage in such cooperative behavior, which allows them to obtain benefits from cooperators while avoiding costs to themselves. It has been shown that the evolution of cooperation can be enhanced if evolutionary dynamics affect group or population sizes [13,14]. Additionally, enhancement of the evolution of cooperation due to rapid evolutionary dynamics is also possible when group size and population size are not affected by evolutionary dynamics. In spatial models where population size and number of interaction partners are fixed, evolutionary dynamics can still impact demographic dynamics and changes in demographic dynamics can subsequently feed back on evolutionary dynamics [15-18]. Evolutionary dynamics can impact demographic dynamics because fitness differences among individuals directly translate into differential reproduction among individuals. The reverse (that demographic dynamics influence evolutionary dynamics) arises because demographic dynamics influence the spatial distribution of cooperators and defectors (which can be characterized by different shapes of clusters of cooperators). The resulting spatial patterns determine assortment between cooperators and defectors, which directly affects fitness and thus evolutionary dynamics [15-18].

However, currently it is not clear how well these spatial models apply to species in which individuals are not embedded in static spatial structures in which each individual has a fixed position and thus a fixed number of interaction partners. For instance, many social insects and mammals live in distinct groups where animals disperse among groups and/or large groups split into several daughter groups [19-28].

Here we use a simulation approach to study the potential effect of rapid evolution in group living animals with fixed population sizes and fixed maximum group sizes. To identify effects of rapid evolution we investigate how assortment of cooperators and defectors and pay-off differences between cooperators and defectors depend on the occurrence of evolutionary dynamics. Specifically we compare two scenarios: (1) a “no selection” scenario in which cooperation does not affect individual fitness, which prevents any effect of evolutionary dynamics on demographic dynamics and (2) a “selection” scenario in which cooperation affects individual fitness, which potentially allows evolutionary dynamics to affect demographic dynamics. The existence and specific effects of rapid evolutionary dynamics can be inferred by comparing the results of both scenarios. Any differences between scenarios can only be explained by the existence of rapid evolutionary dynamics.

## Methods

### Model description

The description of our model is based on the ODD protocol for describing individual- and agent-based models [29].

#### Purpose

The main purpose of this model was to explore whether and how rapid evolutionary dynamics can affect the evolution of cooperative behaviors if migration rates and population size is kept constant.

#### State variables and scales

We assume a population of *N* haploid, asexually reproducing individuals that live in groups of variable size. Each individual has one gene that determines whether it is a cooperator or a defector. In addition, each individual has fitness *F* that determines probabilities of death and reproduction.

#### Process overview and scheduling

Model dynamics proceed in discrete time steps in which five processes occur consecutively: (1) interactions among individuals in a group, which determine individual fitness, (2) fitness-dependent death, (3) fitness-dependent asexual reproduction including mutation, (4) individual migration and (5) splitting of groups that exceed maximum size.

#### Model details

We assume that in each time step all individuals in a group interact with each other, which corresponds to the assumption in spatial models that each individual interacts with all neighbors. To account for differences in group sizes we assumed that in these interactions cooperators pay a total cost *c* and provide a total benefit *b*. If there are *n*_*C*_ cooperators in a group with *n* individuals the pay-off of a cooperator *ρ*_*A*_ is given by:

(1) $$\rho_{A}=\frac{\left(n_{C}-1\right)}{n-1}\cdot b-c$$

and the pay-off of a defector *ρ*_*D*_ is given by:

(2) $$\rho_{D}=\frac{n_{C}}{n-1}\cdot b$$

Note that these pay-offs also correspond to expected pay-off in the case where each cooperator engages in only a single interaction in which a benefit *b* is provided to a randomly chosen group member at a cost *c*.

After receiving pay-offs *ρ*_*i*_, the fitness of an individual *i* is calculated by:

(3) $$F_{i}=1+s\cdot \rho_{i}$$

where *s* is the coefficient of selection. If there is only a single individual in a group no interactions occur and thus these individuals always have a fitness of one.

Death and reproduction are determined by individual fitness where it is assumed that all individuals in the population compete with each other equally (which might arise when groups have overlapping home ranges and rely on the same resources). In each time step ten per cent of all *N* individuals in the population die. Each time the probability *p*_*d*,*i*_ that an individual *i* dies is given by:

(4) $$p_{d,i}=\frac{F_{i}{}^{-1}}{\sum_{j}F_{j}{}^{-1}}$$

where the sum in the denominator includes all individuals that are still alive. Thus, a greater fitness reduces the probability of death.

Reproduction compensates for the loss of dying individuals and thus ensures a constant population size of *N*. We simulate reproduction as a sequence of *N*/10 events in which each time a single individual is chosen to produces a single offspring. The probability *p*_*r*,*i*_ that an individual *i* is selected in each event is given by:

(5) $$p_{r,\;i}=\frac{F_{i}}{\sum_{j}F_{j}}$$

where the sum in the denominator includes all alive individuals excluding newborns. In this case a greater fitness increases the probability of reproduction. We assume asexual reproduction in which the offspring inherits the genome of the parent; and cooperator genes mutate into selfish genes and vice versa with a probability of 0.001.

After reproduction takes place each individual migrates to another randomly selected group with probability *m*. Thereafter groups that exceed a size of *n*_*max*_ individuals split into two daughter groups. This is implemented by creating a new group with half of all group members that are selected randomly. Dispersal that occurs through group splitting is often called budding or propagule dispersal [13,30] and is frequently found in social mammals and social insects [19-27].

#### Model analysis

In the model analysis we compared assortment and pay-off landscapes under scenarios with and without selection. In each scenario we recorded interaction frequencies and mean pay-offs of cooperators and defectors for different frequencies of cooperators and different cost-benefit ratios. In all analyses we assumed a population size *N* of 100. As a baseline setting we assumed a maximal group size *n*_*max*_ of 10, an individual migration rate *m* of 0.02 and no selection (*s* = 0). In additional analyses we varied individual migration rate (*m* = 0.05) and maximum group size (*n*_*max*_ = 5). For each parameter setting we additionally explored the effect of selection (*s* = 0.5).

For each parameter setting we performed simulations for different cost-benefit ratios by keeping costs constant (*c* = 1) and varying benefits. In different analyses we adjusted the investigated range of cost-benefit ratio to focus on the critical cost-benefit ratio up to which cooperators gain greater pay-offs than defectors. The ratio *c*/*b* was varied from 0.01 to 0.99 in steps of 0.01.

For each parameter combination we performed 500 simulations for 100,000 time steps. All simulations were initialized with *N*/*n*_*max*_ groups and *n*_*max*_ individuals per group. In half of the simulations all initial individuals were defectors and in the other half all individuals were cooperators. During simulations we recorded in each time step (after interactions took place) the frequency of cooperators in the population, the frequencies with which cooperators and defectors interact with each other and the mean pay-offs that cooperators and defectors received. Information on pay-offs was used to calculate differences in mean pay-offs. Assortment was calculated by subtracting the average frequency with which cooperators interact with other cooperators and the frequency with which defectors interact with cooperators, which is consistent with the regression definition of relatedness in inclusive fitness models [31]. Note that positive values of this assortment measure mean that cooperators are more likely to be associated with other cooperators, and that negative values mean that cooperators are more likely to be associated with defectors.

## Results

### No selection scenario

**Figure 1:**
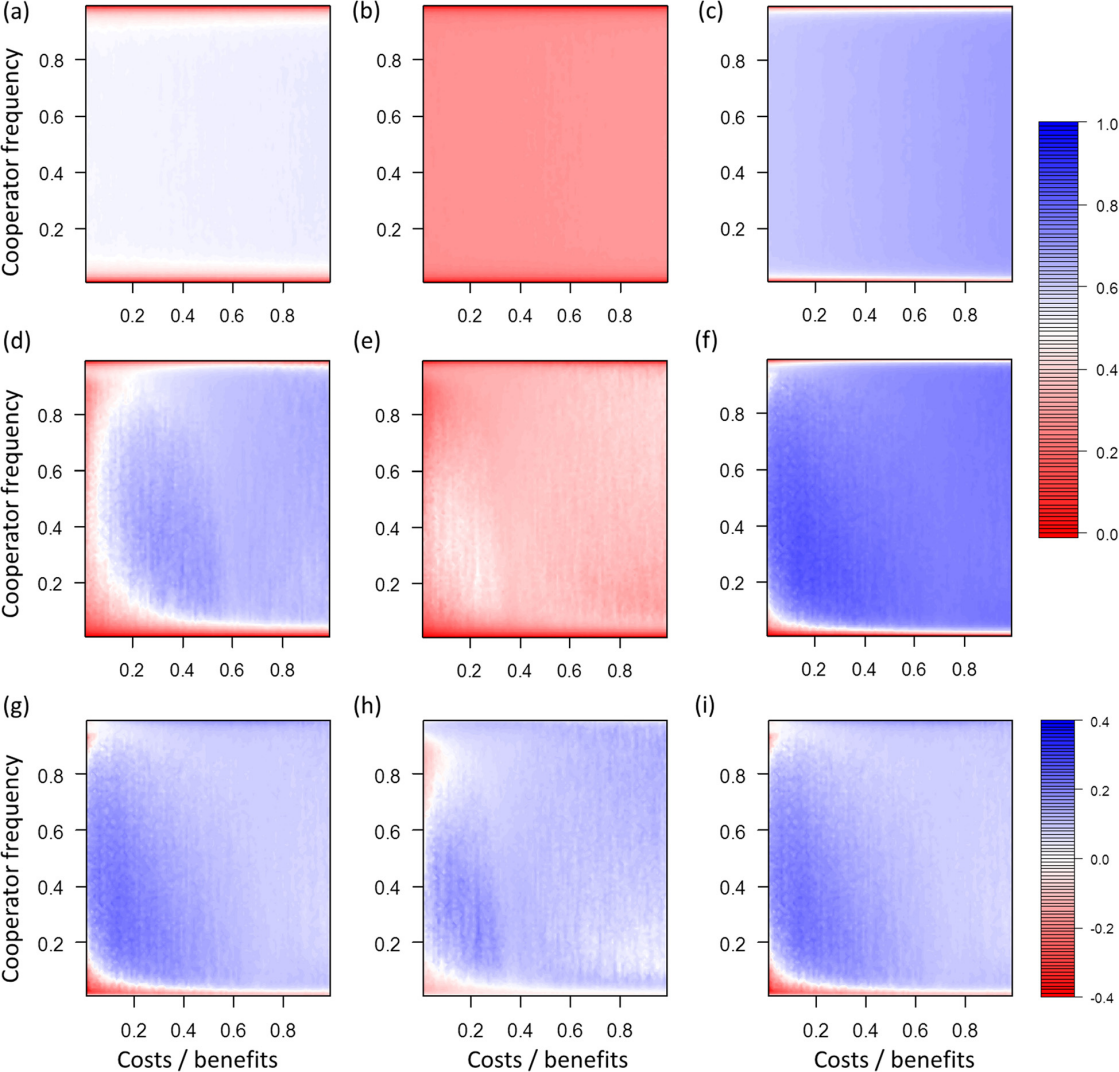
**Assortment at different cost**-**benefit ratios and different frequencies of cooperators.** Blue areas indicate high positive assortment and red values indicate low positive assortment between cooperators. **(a, b, c)** Scenarios without selection (*s* = 0). **(d, e, f)** Matching scenarios with selection (*s* = 0.5). **(g, h, i)** Differences in assortment between scenarios with and without selection. **(a)**, **(d)**, **(g)** Baseline scenario, maximal group size *n*_*max*_ = 10, individual migration rate *m* = 0.02. **(b, e, h)** Increased migration rate (*n*_*max*_ = 10, *m* = 0.05). **(c, f, i)** Decreased group size (*n*_*max*_ = 5, *m* = 0.02).

**Figure 2:**
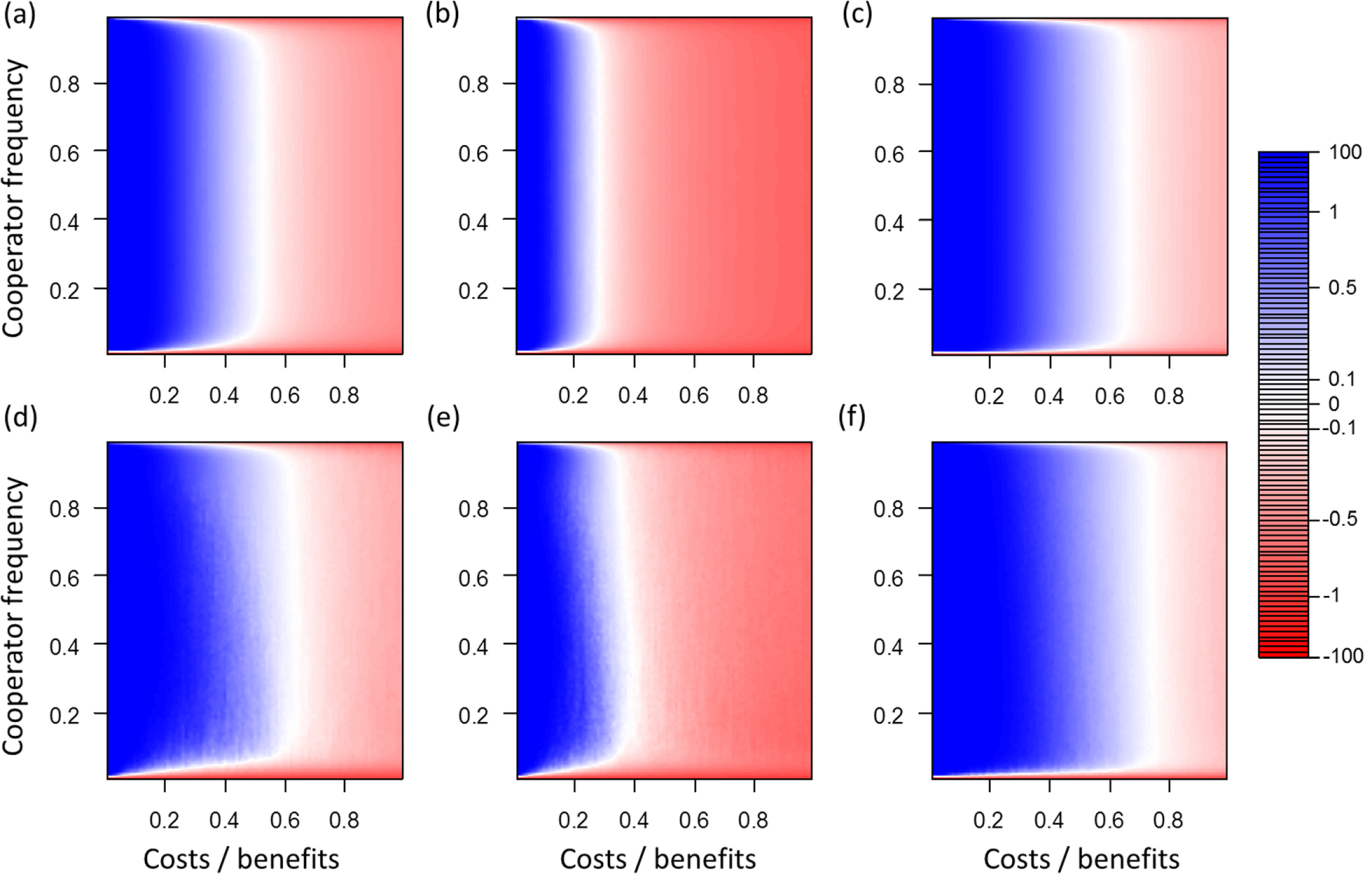
**Differences of mean pay**-**offs of cooperators and defectors at different cost**-**benefit ratios and different frequencies of cooperators.** In blue areas cooperators receive greater average pay-offs and in red areas defectors receive greater average pay-offs. **(a, b, c)** Scenarios without selection (*s* = 0). **(d, e, f)** Matching scenarios with selection (*s* = 0.5). **(a, d)** Baseline scenario, maximal group size *n*_*max*_ = 10, individual migration rate *m* = 0.02. **(b, e)** Increased migration rate (*n*_*max*_ = 10, *m* = 0.05). **(c, f)** Decreased group size (*n*_*max*_ = 5, *m* = 0.02).

In the absence of selection (*s* = 0) assortment and thus pay-off differences between cooperators and defectors are not frequency dependent, except for extreme frequencies where assortment drops and cooperators receive smaller average pay-offs (Figures 1, 2). At very high and very low frequencies of cooperators assortment is very low. The reason for this is most apparent in the case in which there is only a single cooperator in a population of defectors. A single cooperator cannot interact with other cooperators, which means that no assortment of cooperators is possible and therefore a single cooperator also cannot obtain a greater pay-off than the average defector.

Changes in individual migration rates and maximum group sizes affected assortment (Figure 1) and therefore also the critical cost-benefit ratio up to which cooperator can gain greater average pay-offs than defectors (Figure 2). These results emerged because greater migration rates lead to more mixing, which decreases positive assortment and makes it easier for defectors to exploit cooperators. Smaller maximum group sizes lead to smaller average group sizes, which can increase positive assortment because fewer individuals are affected by migration events.

### Selection scenario

In all investigated parameter settings selection markedly changed model dynamics (Figures 1 and 2). For most conditions selection led to an increase in positive assortment. As a consequence, the selection scenarios led to an increase of the critical cost-benefit ratio at which cooperators gained greater pay-offs than defectors. However, in particular for low frequencies of cooperators the opposite effect emerged. Assortment was lower than in the corresponding no selection scenario, which resulted in frequency-dependent effects that act against the evolution of cooperation at small frequencies of cooperator. This frequency-dependent effect widened the adaptive valley that needs to be crossed when cooperators evolve in a population of defectors (see Discussion for a more detailed treatment of the term adaptive valley).

**Figure 3:**
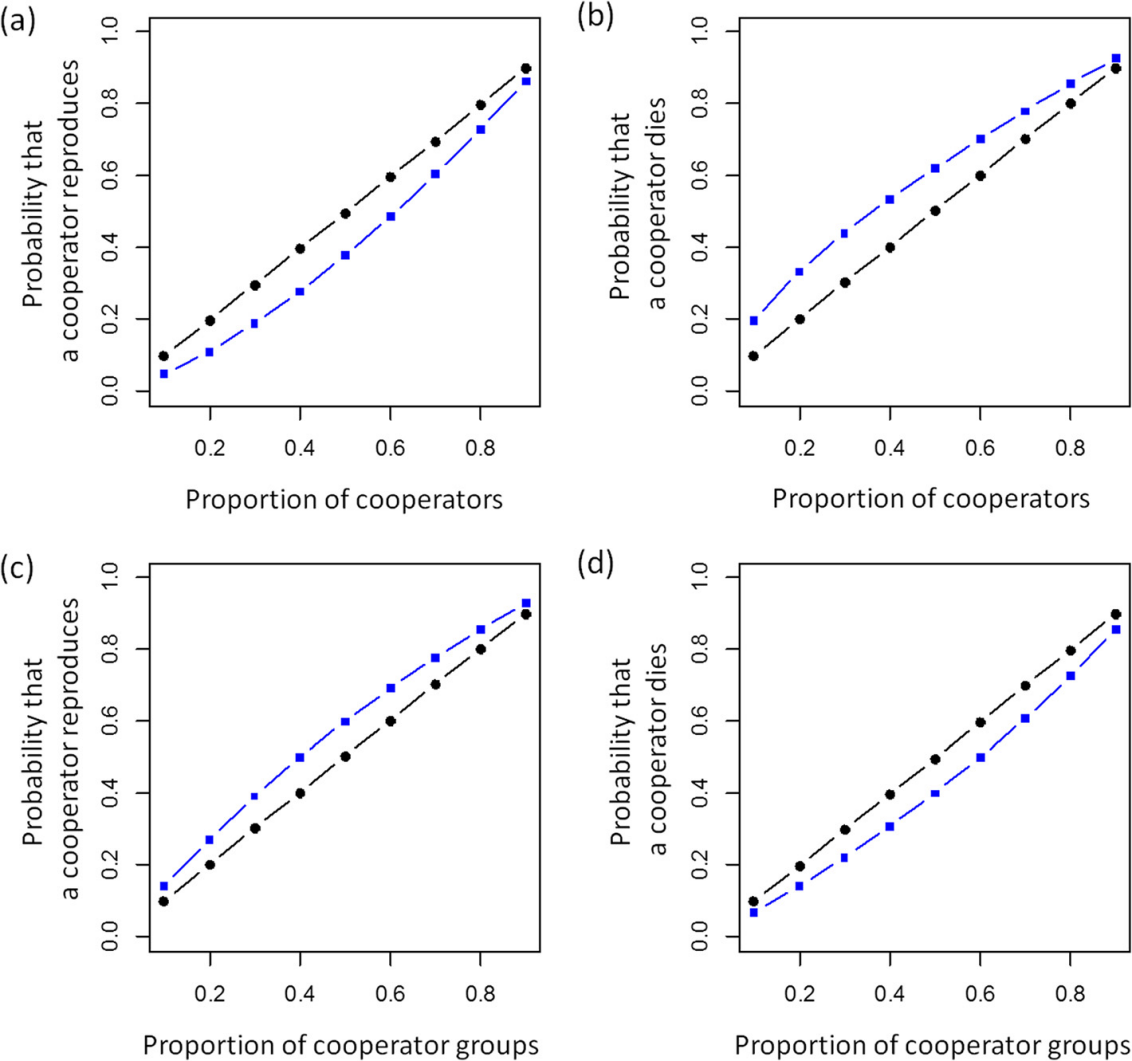
**Illustration of within-group and between-group selection in absence and presence of selection.** In all scenarios we assumed a population that consists of 10 groups of 10 individuals. For within-group selection **(a, b)** we varied the proportion of cooperators within groups while assuming that all groups have the same proportion of cooperators. For between-group selection **(c, d)** each groups contained either only cooperators or only defectors and we varied the proportion of cooperator groups. In **(a)** and **(c)** we assumed that a single individual reproduces and calculated the fitness-dependent probability that the reproducing individual is a cooperator. In **(b)** and **(d)** we assumed that a single individual dies and calculated the fitness-dependent probability that the dying individual is a cooperator. In all cases we assumed costs of 1, and benefits of 2. Circles show results in absence of selection (*s* = 0). Squares show results in presence of selection (*s* = 0.5).

Changes in assortment that occurred due to the presence of selection indicate the existence of rapid evolutionary dynamics. Greater positive assortment can be achieved by rapid evolution because in mixed groups that contain cooperators and defectors, cooperators have higher death and lower reproduction rates than defectors. As shown in Figure 3, in absence of selection, reproduction and death processes occur according to the random expectation where cooperators and defectors do not differ in reproduction and death rates. In presence of selection, changes in reproduction and death probabilities favor defectors in within-group selection and cooperators in between-group selection (Figure 3). For the assumed coefficient of selection (*s* = 0.5), changes in reproduction and death probabilities are rather small, which allows substantial influence of stochasticity. Nevertheless, in selection scenarios the frequency of defectors tends to increase in mixed groups. At the same time, groups with many cooperators tend to increase in size and fission at a faster rate compared to groups with few cooperators. These two processes counteract the mixing process that emerges from migration and thus rapid evolution can increase positive assortment.

Our finding that rapid evolutionary dynamics widened the adaptive valley for the evolution of cooperation shows that rapid evolutionary dynamics do not always increase positive assortment. At low frequencies of cooperators, rapid evolutionary dynamics decreased positive assortment (compare scenarios with and without selection in Figure 1). This happened because selection acts against the formation of groups with many cooperators. At low frequencies of cooperators no or only few groups with many cooperators exist. In addition, individuals that immigrate into such groups are likely to be defectors. In absence of selection it is easily possible that defectors in mixed groups die without reproducing, which increases the frequency of cooperators in such groups. In contrast, when selection takes place such dynamics are less likely because defectors die with lower probability than cooperators. In selection scenarios, groups with many cooperators often emerge by fissioning. However, this only works well if fissioning can counterbalance the immigration of defectors. At low frequencies of cooperators, most immigrants are defectors, which makes it more difficult to maintain groups in which most or all individuals are cooperators.

### Additional analyses

Appendix A contains results from additional analyses, which indicate that the results reported above do not qualitatively change if (1) fitness affects only death or only reproduction (Appendix A), (2) the coefficient of selection is increased to 1 (Appendix A), and (3) mutation rate is decreased or increased (Appendix A). Additional examples show how assortment changes with the frequency of cooperators within single simulation runs (Appendix A). Appendix B contains the description and analysis of an extended version of the model in which individuals reproduce sexually.

## Discussion

Analyses of our model showed that rapid evolution can crucially influence the evolution of cooperation in group-living individuals even when population size and maximum group size is fixed (Figures 1, 2). Rapid evolutionary dynamics emerge because selection pressures allow evolutionary dynamics to occur on the same timescale as demographic dynamics. In the “no selection” scenarios birth and death processes are not influenced by evolutionary dynamics and thus cooperators and defectors do not differ in survival and reproduction rates. In contrast, in the “selection” scenarios evolutionary dynamics directly influence survival and reproduction rates (Figure 3). This influences positive assortment, which creates a closed feedback between evolutionary and demographic dynamics on the same timescale. Thus, in our model dynamics emerge that are similar to those that have been observed in spatial models where individuals are embedded in static spatial structures in which each individual has a fixed position and thus a fixed number of interaction partners [15-18].

The increase in positive assortment that occurs in our model results from rapid evolutionary dynamics. This has some similarities to dynamics generated by cooperation models that allow individuals to change their interaction partners [32-35]. Such changes can facilitate the evolution of cooperation when social network dynamics lead to the preferential formation of connections between cooperators. This increases positive assortment between cooperators and defectors, which in turn facilitates the evolution of cooperation. In our model, a preferential association of cooperators can also emerge. However, in our model this association is driven by rapid evolutionary dynamics instead of resulting from individual-level decision rules. Because rapid evolutionary dynamics can generate similar dynamics as individual-level decision rules, in some cases such rules might be redundant and rapid evolution could prevent the evolution of these rules. In particular, this might happen if the execution of these rules is costly, e.g. because they require additional cognitive capacities (see [36] for a similar example in the context of social learning).

Previous analyses of rapid evolution of cooperation that assume fixed population sizes often focused on identifying whether rapid evolution enhances or suppresses the evolution of cooperation, while potential density dependent-effects are usually ignored [15-18]. In our analysis we found that rapid evolution does not always facilitate the evolution of cooperation. Instead frequency-dependent effects emerge. This happens because at low frequencies of cooperators within-group dynamics dominate the impact of evolution on demographic dynamics. This decreases positive assortment of cooperators and defectors, which acts against the evolution of cooperation. In contrast, at high frequencies of cooperators between-group dynamics increasingly impact how evolution influences demographic dynamics. This can result in an increase in positive assortment, which favors the evolution of cooperation. Thus, rapid evolutionary dynamics can increase positive assortment which facilitates the evolution of cooperation. The effects of evolution on demographic dynamics in our model are relatively small (Figure 3), which emphasizes that effects of selection on reproduction and survival rates do not have to be extreme in order to critically influence positive assortment and evolutionary dynamics.

The contrasting dynamics at low and high frequencies of cooperators lead to frequency-dependent fitness of cooperators, which creates an adaptive valley for the evolution of cooperation. This means that for several cost-benefit ratios cooperators are expected to gain higher pay-offs than defectors when the frequency of cooperators in the population is already high, but lower pay-offs are expected when the frequency of cooperators is low. In this case, when cooperators are rare, selection pressures lead to a decrease in frequency of cooperators (red areas in Figure 2). In contrast, when cooperators are more common the direction of selection changes and selection pressures lead to an increase in frequency of cooperators (blue areas in Figure 2). In other words, in order to evolve cooperation in a population of defectors the frequency of cooperators has to increase while selection initially acts against this increase until a critical point from which on selection favors cooperation. The increase in frequency of cooperators might be pictured as crossing an adaptive valley.

In particular, positive frequency-dependent selection of cooperative behaviors, which might emerge from rapid evolution, could be an important explanation for species differences in cooperative behaviors. Positive frequency-dependent selection might maintain cooperation in one species and defection in another species even if both species have identical population structures and live in identical environments (i.e. face the same cost-benefit ratio). In other words, similar species could be stuck on either side of an adaptive valley that is created by positive frequency dependence. This would be for instance possible if an ancestor of one but not the other species managed to cross this valley. Although crossing adaptive valleys should be generally unlikely, such events might be promoted by population expansion [37,38], disturbances that result in population bottlenecks [39], stochastic processes [40] or a combination of these and other processes.

As an example, an adaptive valley might explain difference in cooperative behaviors in humans and chimpanzees. Recently, Langergraber et al. [41] showed that humans and chimpanzees have comparable levels of genetic between-group differentiation, which contradicts previous arguments that extensive levels of human cooperation are explained by particularly high genetic differentiation among human groups [42,43]. Positive frequency-dependent selection could potentially explain why humans nevertheless show much more cooperation. At least positive frequency-dependent selection could explain why differences in cooperation are maintained and why humans and chimpanzees do not converge to similar levels of cooperation.

## Conclusions

Analyses of our model showed that rapid evolutionary dynamics can crucially influence the evolution of cooperation in group-living individuals and lead to positive frequency-dependent selection. The observed dynamics emerge because selection pressures allow evolutionary and demographic dynamics and, consequently, also feedback between assortment and evolution to occur on the same timescale. In particular, emerging positive frequency-dependent selection could be an important explanation for differences in cooperative behaviors among different species with similar population structures such as humans and chimpanzees.

## Appendix A: Additional analyses

**Figure 4:**
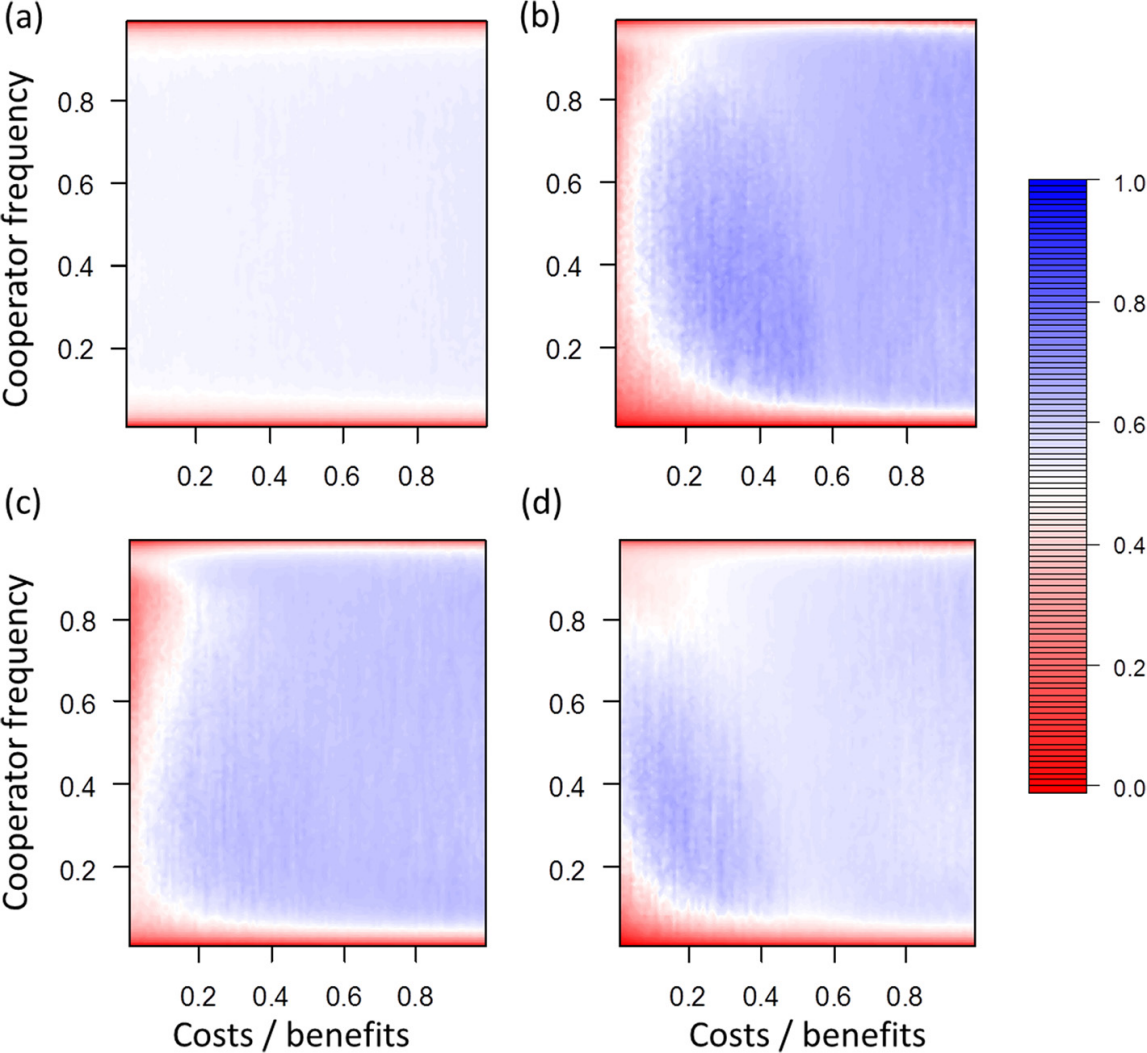
**Fitness affects only death or reproduction.** Assortment at different cost-benefit ratios and different frequencies of cooperators in the baseline scenario (*n*_*max*_ = 10, *m* = 0.02). **(a)** No selection (*s* = 0). **(b)** Fitness affects birth and death (*s* = 0.5). **(c)** Fitness affects only death (*s* = 0.5). **(d)** Fitness affects only reproduction (*s* = 0.5).

**Figure 5:**
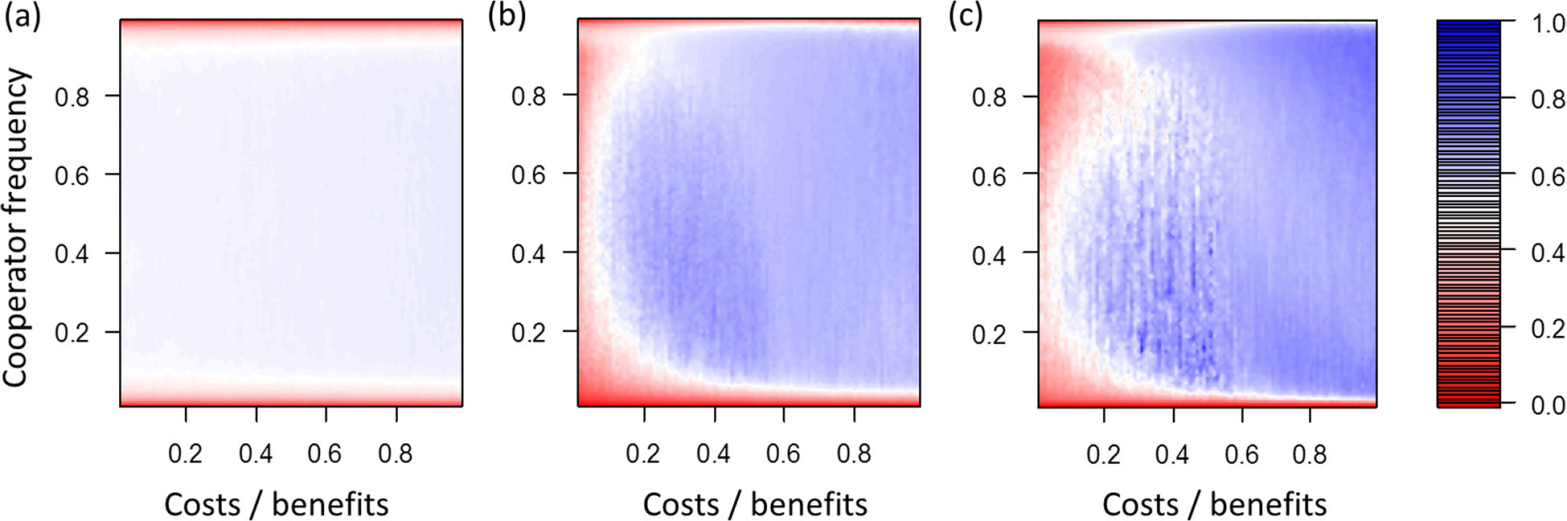
**Changes in the coefficient of selection *s*.** Assortment at different cost-benefit ratios and different frequencies of cooperators in the baseline scenario (*n*_*max*_ = 10, *m* = 0.02) with *s* = 0 **(a)**, *s* = 0.5 **(b)** and *s* = 1 **(c)**.

**Figure 6:**
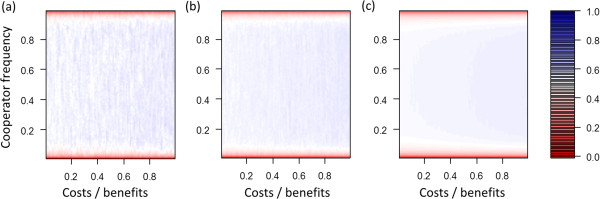
**Changes in mutation probabilities.** Assortment at different cost-benefit ratios and different frequencies of cooperators in the baseline scenario (*n*_*max*_ = 10, *m* = 0.02) with mutation probability set to 0.000001 **(a)**, 0.0001 **(b)** and 0.01 **(c)**.

**Figure 7:**
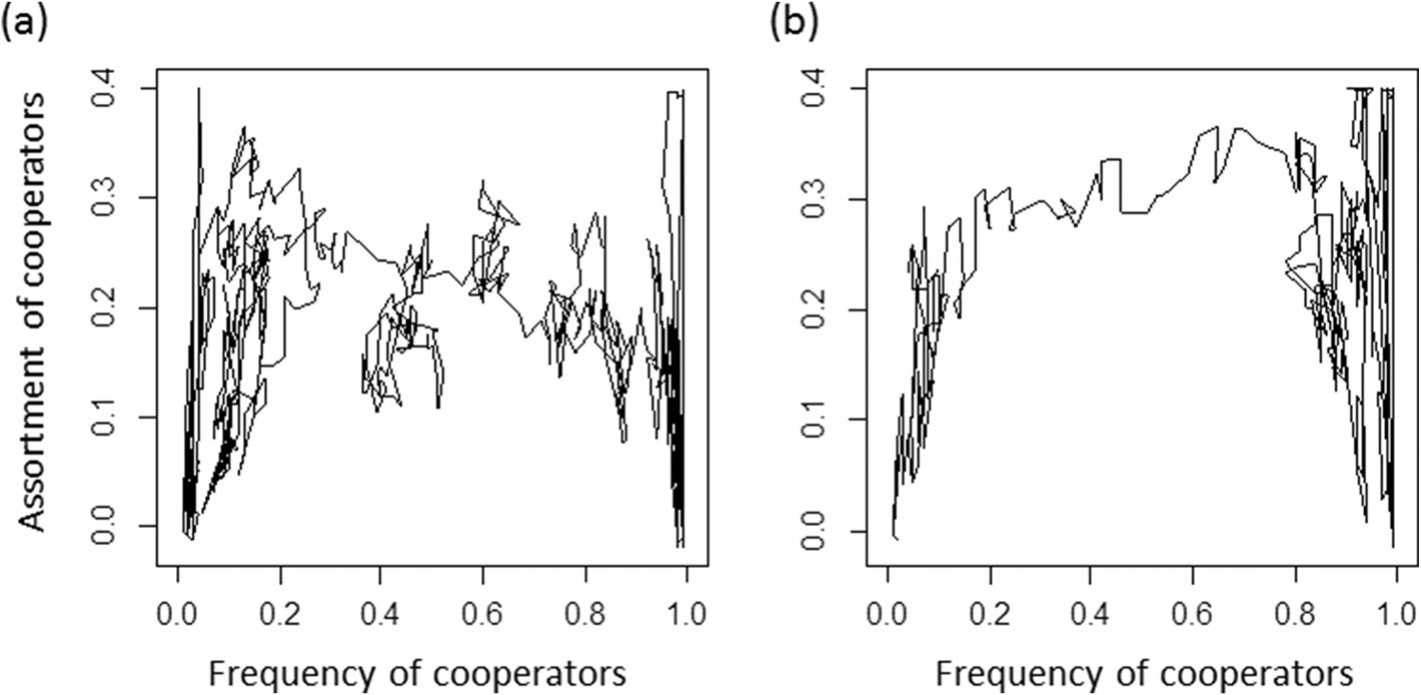
**Examples of how assortment changes with the frequency of cooperators within single simulation runs. (a)** Baseline scenario without selection (*s* = 0). **(b)** Baseline scenario with selection (*s* = 0.5). In both cases cost-benefit ratios were set to 0.5, simulations were initialized with a population of defectors and depicted dynamics comprise 25,000 simulated time steps.

Additional analyses indicate that the results reported in the main text do not qualitatively change if (1) fitness affects only death or only reproduction (Figure 4), (2) the coefficient of selection is increased to 1 (Figure 5), and (3) mutation rate is decreased or increased (Figure 6). Figure 7 contains examples that show how assortment changes with the frequency of cooperators within single simulation runs.

## Appendix B: Sexual reproduction model

We extended the model described in the main text to explore the influence of sexual reproduction. We now assume a population of diploid organisms, *N*_*f*_ females and *N*_*m*_ males. Each time step *N*_*f*_/10 females and *N*_*m*_/10 males die and then *N*_*f*_/10 females and *N*_*m*_/10 males are born. During reproduction an individual inherits one allele from the father and one from the mother. Heterozygous individuals become cooperators with 50% chance (i.e. one of the two alleles is silenced randomly). Females are assumed to be philopatric and thus only disperse by group fissioning but not individually. Fissioning is implemented as described in the main text. However, now only the number of females in a group determines whether a group splits. We also focus on cooperation among females and thus assume that only females interact with each other. Thus only females gain fitness as described in the main text and only female death and reproduction is fitness-dependent. Males do not interact with each other and thus all have the same fitness. Therefore also death and reproduction of males occurs randomly. In addition, males are assumed to repeatedly migrate individually. However, migration is not explicitly modeled. Instead each time a females reproduces one randomly selected male is assumed to be the father (which implicitly assumed a high individual migration rate of males).

In the analysis of the sexual reproduction model we set the number of females *N*_*f*_ and males *N*_*m*_ to 100, the maximum females groups size *n*_*max*_ to 5. Again we contrasted a scenario without selection (*s* = 0) to a scenario with selection (*s* = 0.5).

**Figure 8:**
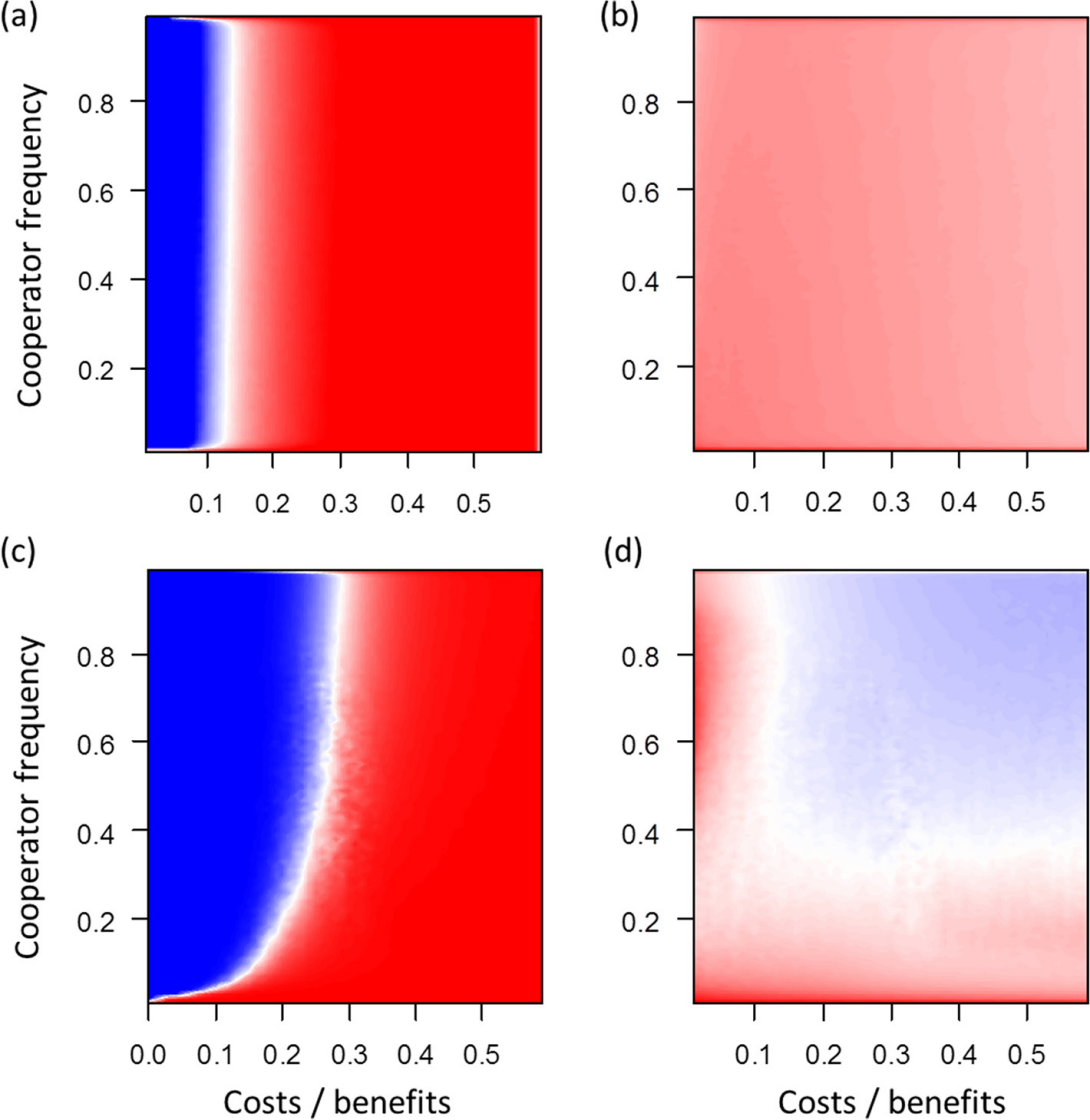
**Differences of mean pay-offs of cooperators and defectors and assortment in the sexual reproduction model. (a, c)** In blue areas cooperators receive greater average pay-offs and in red areas defectors receive greater average pay-offs. **(b, d)** Blue areas indicate high positive assortment and red values indicate low positive assortment cooperators. **(a, b)** Scenario without selection (*s* = 0). **(c, d)** Scenarios with selection (*s* = 0.5). Note that results are shown only for cost-benefit ratios up to 0.6.

Analysis of the sexual reproduction model revealed similar results to those found in the asexual model (Figure 8). However, in the sexual reproduction model a much broader adaptive valley emerged. This happened because reproduction is directly connected to male migration. At low frequencies of cooperators most males carry one or two defector alleles. In this case, males introduce defector alleles into cooperator groups, which increases mixing of cooperator and defectors. Thus, compared to the asexual model it is much more difficult to maintain groups with many cooperators when the frequency of cooperators is low. The situation is very different at high frequencies of cooperators when males are more likely to carry cooperator alleles. In this case, it is less likely that males introduce defector alleles into cooperator groups. This makes it more likely that these groups grow and fission, which leads to an elevated level of positive assortment.

## Competing interests

The authors declare that they have no competing interests.

## Authors’ contributions

MF conceived of the study, participated in the study design, developed the model, performed model analyses and participated in drafting the manuscript. OS and JO participated in the design of the study and in drafting the manuscript. All authors read and approved the final manuscript.
